# Lack of allele-specific efficacy of a bivalent AMA1 malaria vaccine

**DOI:** 10.1186/1475-2875-9-175

**Published:** 2010-06-21

**Authors:** Amed Ouattara, Jianbing Mu, Shannon Takala-Harrison, Renion Saye, Issaka Sagara, Alassane Dicko, Amadou Niangaly, Junhui Duan, Ruth D Ellis, Louis H Miller, Xin-zhuan Su, Christopher V Plowe, Ogobara K Doumbo

**Affiliations:** 1 Malaria Research and Training Center, Department of Epidemiology of Parasitic Diseases, Faculty of Medicine, Pharmacy and Dentistry, Bamako, Mali; 2 Laboratory of Malaria and Vector Research, NIAID, NIH 12735 Twinbrook Parkway, Twinbrook III, Room 3E-32C, Bethesda, MD 20892-8132, USA; 3 Howard Hughes Medical Institute/Center for Vaccine Development, University of Maryland School of Medicine, 685 West Baltimore Street HSF1-480 Baltimore, MD 21201, USA; 4 Laboratory of Malaria Immunology and Vaccinology, National Institute of Allergy and Infectious Diseases (NIAID), National Institutes of Health, 5640 Fishers Lane, Twinbrook I, Room 1109 Rockville, MD 20852, USA; 5 Department of Public Health, Faculty of Medicine, Pharmacy and Dentistry, Bamako, Mali

## Abstract

**Background:**

Extensive genetic diversity in vaccine antigens may contribute to the lack of efficacy of blood stage malaria vaccines. Apical membrane antigen-1 (AMA1) is a leading blood stage malaria vaccine candidate with extreme diversity, potentially limiting its efficacy against infection and disease caused by *Plasmodium falciparum *parasites with diverse forms of AMA1.

**Methods:**

Three hundred Malian children participated in a Phase 2 clinical trial of a bivalent malaria vaccine that found no protective efficacy. The vaccine consists of recombinant AMA1 based on the 3D7 and FVO strains of *P. falciparum *adjuvanted with aluminum hydroxide (AMA1-C1). The gene encoding AMA1 was sequenced from *P. falciparum *infections experienced before and after immunization with the study vaccine or a control vaccine. Sequences of *ama1 *from infections in the malaria vaccine and control groups were compared with regard to similarity to the vaccine antigens using several measures of genetic diversity. Time to infection with parasites carrying AMA1 haplotypes similar to the vaccine strains with respect to immunologically important polymorphisms and the risk of infection with vaccine strain haplotypes were compared.

**Results:**

Based on 62 polymorphic AMA1 residues, 186 unique *ama1 *haplotypes were identified among 315 *ama1 *sequences that were included in the analysis. Eight infections had *ama1 *sequences identical to 3D7 while none were identical to FVO. Several measures of genetic diversity showed that *ama1 *sequences in the malaria vaccine and control groups were comparable both at baseline and during follow up period. Pre- and post-immunization *ama1 *sequences in both groups all had a similar degree of genetic distance from FVO and 3D7 *ama1*. No differences were found in the time of first clinical episode or risk of infection with an AMA1 haplotype similar to 3D7 or FVO with respect to a limited set of immunologically important polymorphisms found in the cluster 1 loop of domain I of AMA1.

**Conclusion:**

This Phase 2 trial of a bivalent AMA1 malaria vaccine found no evidence of vaccine selection or strain-specific efficacy, suggesting that the extreme genetic diversity of AMA1 did not account for failure of the vaccine to provide protection.

## Background

Combined with other measures, vaccination is considered a promising approach to control and eventually eliminate malaria [1,2]. Extensive polymorphism in many *Plasmodium falciparum *proteins may limit the efficacy of vaccines based on just one or two allelic variants that are not broadly cross-protective against diverse antigens found in natural infections. For example, a multi-antigen blood stage malaria vaccine evaluated in Papua New Guinea reduced parasite density and prevalence of infection in a strain-specific manner, suggesting selection of non-vaccine variants [3]. In contrast, immunization with RTS,S, a vaccine directed against the pre-erythrocytic circumsporozoite protein [4], does not appear to result in selection or allele-specific efficacy [5,6].

Apical membrane antigen-1 (AMA1) is a leading blood stage malaria vaccine candidate that is thought to play a critical role in erythrocyte invasion. Antibodies against AMA1 have been shown to block parasite invasion [7-11], and sero-epidemiological studies have shown an association of anti-AMA1 antibodies with naturally acquired protection against malaria [12,13]. Animal studies [14] have shown the ability of vaccines based on this antigen to stimulate antibody responses that were correlated with a reduction of parasite density.

AMA1 is expressed mainly during the *P. falciparum *asexual or blood stage, and examination of *ama1 *sequences from natural *P. falciparum *infections has shown extreme diversity in this gene. There are up to 62 polymorphic amino acid sites in AMA1, representing more than 15% of the amino acid sites distributed over three domains of the protein [15,16]. The greatest polymorphism is seen in domain I, especially in a cluster of amino acids near a hydrophobic pocket [17] that is thought to play a role in erythrocyte invasion, the cluster 1 loop of domain I (c1L), which includes amino acid residues 196, 197, 199, 200, 201, 204, 206 and 207 [18]. Based on antigenic escape modeling, growth and invasion inhibition assays [19] and molecular epidemiological studies of the impact of AMA1 polymorphism on risk of clinical malaria [16], c1L has been identified as a key target of both strain-specific antibodies and allele-specific naturally acquired protective immune responses.

The malaria vaccine AMA1-C1 is a bivalent vaccine comprised of recombinant AMA1 based on the 3D7 and FVO reference strains of *P. falciparum *expressed in *Pichia pastoris *and adjuvanted with aluminum hydroxide, developed by the Malaria Vaccine Development Branch of the National Institute for Allergy and Infectious Diseases of the U.S. National Institutes of Health and tested at the Malaria Research and Training Center at the University of Bamako in Mali. Phase 1 studies of AMA1-C1 in Malian adults and children showed that it was acceptably safe and tolerable and modestly immunogenic [20,21]. However, as with other vaccine antigens, the high degree of polymorphism observed in this protein [16,22-24] and possible strain specificity of the immune response [25] may limit its success as a vaccine candidate. Since failures of AMA1 vaccine in animal models have been attributed to the diversity of the antigen used in vaccine formulation [26,27], careful measurement of allele-specific efficacy and vaccine selection are important components in assessing the efficacy of malaria vaccine candidates in field trials conducted in endemic countries [28].

A Phase 2 clinical trial of AMA1-C1 vaccine in Malian children showed no impact of vaccination on parasite density or clinical malaria [29]. Even in the absence of measurable overall parasitological or clinical efficacy, it was hypothesized that children who received the AMA1 vaccine might have a decreased incidence of clinical malaria caused by parasites having AMA1 alleles similar to the vaccine alleles as a consequence of the strain specificity of the immune response. In this scenario, the low vaccine efficacy might be explained by the vaccine only providing protection against parasites with AMA1 haplotypes (based on the immunologically relevant polymorphic amino acid residues in c1L) similar to those of the strains represented in the vaccine formulation.

Data and samples collected during this trial were used to assess whether this bivalent malaria vaccine produced a response that was specific to the vaccine antigens, resulting in selection of alleles differing from the vaccine strain. It was further reasoned that even if selection could not be demonstrated directly by comparing frequencies of vaccine-type and non-vaccine type AMA1 in vaccinees and controls, these data might provide indirect evidence for allele-specific immunity elicited by the vaccine.

## Methods

### Overall study design

Samples used in this study were collected during a double-blind, controlled, Phase 2 trial of the safety and efficacy of the AMA1-C1 malaria vaccine conducted in Bancoumana, Mali from 2006 to 2007. The main objective of the study was to compare the protection induced by a bivalent AMA1 vaccine composed of 3D7 and FVO strains and a *Haemophilus influenza *B vaccine control (Hiberix^®^, GlaxoSmithKline, Uxbridge, UK). Details on the methods and overall results of the trial are described elsewhere [20,29]. Briefly, 300 children aged from 2 to 3 years inclusive, were randomly assigned on a 1:1 basis to the vaccine or control group. Two doses of vaccine were given to patients at 28 days intervals. Immunized children were followed for up to 52 weeks. Follow up for vaccine efficacy started 14 days after the last immunization (day 42) and extended to Day 156. Procedures included monthly clinical examination and malaria smears, and blood collection on filter paper for malaria parasite typing. Those presenting with clinical symptoms outside scheduled visits also had malaria smears and blood collected on filter paper for malaria parasite typing.

### Site and sample collection

**Figure 1:**
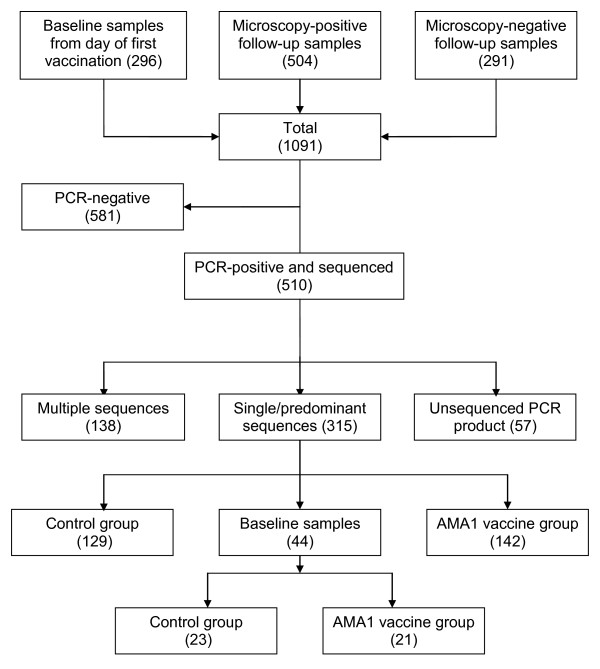
**Flow chart showing the samples and apical membrane antigen-1 (AMA1) ectodomain sequences used in the analysis**.

Samples were collected at the Malaria Research and Training Center research clinic in Bancoumana, a rural village 60 km southwest of Bamako in Mali, West Africa. The health district of Bancoumana has a population of approximately 22,000 inhabitants. Malaria transmission is hyper-endemic and seasonal (June-November), with peak transmission in October. The blood collection procedure onto filter paper was performed as described by Duan *et al *[30]. The study was approved by institutional review boards of the Faculty of Medicine, Pharmacy, and Dentistry of the University of Bamako and the National Institute of Allergy and Infectious Diseases, and written informed consent was obtained from parents or guardians of study participants prior to inclusion in the study. Three hundred blood sample filter papers were collected the day of first vaccination and served as a baseline time point in this study. In addition, 504 infections, as determined by thick smear microscopy, were collected after vaccination (day 42 to day 156). Finally, 291 thick smear-negative samples from the same period of observation were randomly selected from the study database (Figure 1) in hopes of capturing episodes of sub-patent parasitaemia.

### DNA extraction

Malaria parasite DNA was extracted using the QiaAmp 96 DNA blood kit (Qiagen, Valencia CA) according to the manufacturer's instructions.

### Ama1 gene amplification and sequencing

A nested PCR was used to amplify the entire ectodomain coding sequence of *ama1 *(1861 bp), using primers designed by Duan *et al *[30]. Briefly, a nested PCR was performed using primers F2 (tcaaattaatgtacttgtta) and R8 (ttttagcataaaagagaagc) with the following cycling conditions: initial DNA denaturation at 94°C for 2 min, followed by 38 cycles of denaturation at 94°C for 20 sec; primer annealing at 52°C for 10 sec, and extension at 60°C for 3 min. A final extension was conducted at 60°C for 5 min. PCR products from the first amplification were used as the template for a second round of amplification with primers n1 (atgagaaaattatactgcgt) and n2 (tgattatatcagacgttgaa). Amplification conditions were exactly the same as in the primary amplification only with 30 cycles instead of 38. Each primary PCR reaction contained a total volume of 27 μl with 22.5 μl of Platinum super mix in 96-well plates (Invitrogen, Carlsbad, CA), 1 μl of each primer and 2.5 μl of malaria parasite DNA. The secondary PCR used 45 μl of Platinum Mix, 1 μl of each primer and 5 μl of primary PCR product. PCR products were electrophoresed on a high-throughput pre-cast gel system (Invitrogen, Carlsbad, CA) then visualized and photographed under ultraviolet light. Amplified products were purified using Exo-SAP-it (US Biomedical, Cleveland, Ohio). Purified products were sequenced using primers described by Duan et al. [30] on an ABI 3730XL automatic sequencer (Applied Biosystems, Foster City CA). Sequences were deposited in GenBank [GenBank HM562354-HM562668].

### Sequence alignment, statistical and genetic analysis

DNA sequences were analysed using Sequencher 4.8 software (Gene Codes Corporation, MI). A sequence was considered to be from a multiple-allele infection when the secondary peak height was 50% or more of the primary peak height at any polymorphic site. Analyses were initially performed on the 1206 bp region covering domain 1 to domain 3 [30] and then limited to cluster one and one loop (c1L) as described by Dutta *et al *based on antigenic escape residues [19]. Reference sequences were the 3D7 [GenBank AF512508] and FVO strains [GenBank AF277003]. Because haplotypes could not be determined for multiple-allele infections, only sequences classified as corresponding to single or predominant infections with secondary peak height less than 50% of the primary peak at polymorphic codons were used for this analysis.

**Figure 2:**
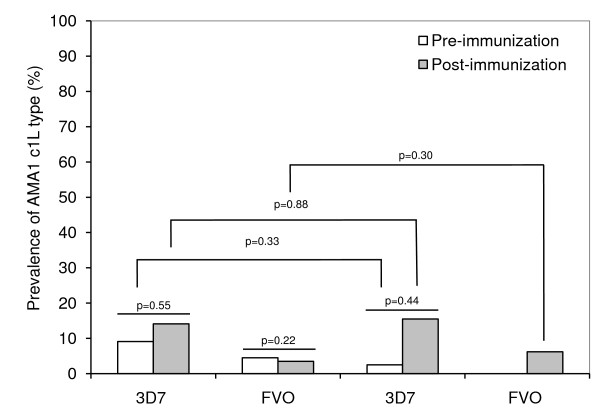
**Frequency of 3D7 and FVO apical membrane antigen-1 (AMA1) cluster 1 loop (c1L) haplotypes observed before and after immunization in the AMA1-C1 vaccine and control groups**.

Parasite population genetic parameters were estimated using MEGA 4.50.3 [31] and DNASP 4.50.3 [32] software. The parameters of interest included: nucleotide diversity (π), which is a measure of the average number of nucleotide differences per site between any two DNA sequences chosen randomly from the sampled population; haplotype diversity (Hd), measuring the probability of randomly choosing two individuals bearing different haplotypes; the average number of nucleotide differences (k); the number of net nucleotide substitutions between populations (D_a_); the average number of nucleotide substitutions per site (D_xy_); the average number of nucleotide differences (k); and Tajima's test, which estimates departures from neutrality by comparing an estimate of the number of segregating sites to the average number of mutations between pairs in the sample. Groups of sequences were also compared by assessing changes in allele frequencies and DNA divergence between populations. In addition, vaccine and control groups were compared, before and after vaccination (Figure 2), with the reference sequences 3D7 and FVO to assess any change in the sequence distribution after vaccination.

AMA1 haplotypes were assigned to haplotype groups (or subpopulations) using a Bayesian clustering algorithm implemented in STRUCTURE software [33]. The model conditions have been described previously [30], and consisted of running ten runs of 50,000 burn-ins and 100,000 iterations were performed for K = 1 to 10 using admixture models.

A malaria clinical episode was defined as axillary temperature > 37.5°C and a parasitaemia greater than or equal to 2,500 asexual *P. falciparum *parasites per mm^3 ^[34]. SAS 9.1.3 statistical software (Cary, NC) was used to assess the time to malaria first clinical episode with a 3D7 or FVO c1L haplotype as defined by Dutta [19] and Takala [16]. Cox proportional hazards regression was used to model the association between study group and risk of a clinical episode with parasites having an AMA1 c1L haplotype identical to 3D7 or FVO. Chi square tests were used to compare the frequency of c1L haplotypes in the two treatment arms.

## Results

### Measures of selection and allele-specific efficacy

Of 1,091 samples subjected to PCR amplification of *ama1*, 510 were PCR-positive and 453 of these yielded full *ama1 *sequences (Figure 1). Of these, 315 were classified as single/predominant allele infections and were included in the haplotype analysis while 138 classified as multiple-allele infections were excluded from haplotype analyses. Table 1 shows the number of *ama1 *haplotypes in each treatment group at baseline and during the efficacy observation period. Among 315 *ama1 *sequences, 186 unique amino acid haplotypes were identified with haplotype prevalences ranging from 0.3% to 2.54% confirming the extreme diversity in AMA1 found at another site in Mali [16].

**Table 1:** Genetic diversity in the gene encoding apical membrane antigen-1 (AMA1) before and after immunization with a bivalent AMA1 malaria vaccine

	Number of sequences	Number of haplotypes	Haplotype diversity (Hd)	Nucleotide diversity (π)	Average number of nucleotide differences (k)	Tajima D	Tajima D p-value
Control group							
Baseline	23	22	0.996	0.0163	19.672	0.3308	> 0.1
Post-immunization	129	96	0.993	0.0172	20.729	1.4110	> 0.1
Control total	152	112	0.994	0.0179	20.726	0.9110	> 0.1
AMA1 vaccine group							
Baseline	21	21	1.000	0.0176	21.210	0.2413	> 0.1
Post-immunization	142	94	0.989	0.0172	20.773	1.8010	> 0.05
AMA1 vaccine total	163	106	0.990	0.0172	20.792	1.0150	> 0.1

Based on the entire set of polymorphic sites within the AMA1 ectodomain (domains I, II and III), there were eight infections with an AMA1 haplotype identical to 3D7 among the 315 analysed, all of which were detected in samples from the post-immunization efficacy observation period; there were no 3D7-identical sequences in infections detected among the 44 samples collected at baseline, before immunization. No AMA1 sequences exactly matched the haplotype of the vaccine strain FVO. Of the eight 3D7-type sequences detected during the post-vaccination efficacy observation period, 5/142 (frequency of 3.5%) were in the AMA1 malaria vaccine group and 3/129 (frequency of 2.3%) were in the control group with a Fisher's exact p-value of 0.72. Thus there was no suggestion that allele-specific vaccine selection resulted in a decreased frequency of infections harboring parasites with 3D7-type alleles with respect to the whole AMA1 ectodomain.

Based only on the c1L haplotype, 14.3% sequences matched the 3D7 vaccine strain while 4.8% matched FVO, similar to the higher prevalence of 3D7-type AMA1 c1L at another vaccine testing site in Mali [16]. While there was a slightly higher proportion of both 3D7 and FVO-type AMA1 c1L haplotypes in the control group at baseline before vaccines were administered, this difference was not significant, and there were no significant differences in the proportions of 3D7 and FVO c1L haplotypes when comparing pre-immunization to post-immunization *P. falciparum *clinical episodes (table 2) and infections (Figure 2) in either the malaria vaccine or control groups.

**Table 2:** Incidence of malaria clinical episodes with apical membrane antigen-1 (AMA1) cluster 1 loop (c1L) haplotype matching vaccine strains

Strain and vaccine group	Vaccine strain c1L type	Non-vaccine strain c1L type	Incidence	p-value
3D7 strain				
Control vaccine	12	27	30.8%	0.20
AMA1-C1	8	35	18.6%	
FVO strain				
Control vaccine	3	32	8.6%	0.55
AMA1-C1	3	40	7.0%	

#### Haplotype diversity before and after immunization

To assess whether immunization with AMA1-C1 resulted in decreased *ama1 *diversity, *ama1 *sequences from infections present before immunization were compared to those occurring after immunization. The hypothesis was that if vaccination reduced the risk of infection caused by parasites with AMA1 similar to the vaccine strains, the AMA1 haplotype frequency of infecting strains would be reduced, as measured by reduced diversity parameters in the malaria vaccine group compared to controls following immunization.

Immunization with the AMA1-C1 vaccine had no effect on various measures of genetic diversity. At baseline the two groups were comparable, with 21 haplotypes among 21 infections in the vaccine group and 22 haplotypes among 23 infections in the control group, and with similar values of Hd and π in both groups (Table 1). The average number of nucleotide differences between the two groups at baseline was 20.805, while the average number of nucleotide substitutions per site between the two groups (D_xy_) was 0.0172 and the net nucleotide substitutions between groups (D_a_) was 0.0003 (Table 3). When comparing all post-immunization infections in the vaccine and control groups, haplotype and nucleotide diversity were again very similar (Table 1). Malaria vaccine group and control group sequences were also virtually identical with respect to the number of net nucleotide substitutions per site after vaccination with D_a _value of -0.00002. Moreover, comparing baseline sequences to post-vaccination sequences within the malaria vaccine and control groups, genetic diversity parameters (Hd, π, D_a _and k) were also similar (Table 3). These analyses do not provide evidence of a reduction in *ama1 *diversity due to the malaria vaccine during the follow up period.

**Table 3:** Comparison of genetic divergence between apical membrane antigen-1 (AMA1) sequences *P. falciparum *infections occurring in children immunized with a bivalent AMA1 malaria vaccine or control vaccine, before and after immunization, and in comparison with the vaccine strains 3D7 and FVO

	Average number of nucleotide differences (k)	Average number of nucleotide substitutions per site (D_xy_)	Number of net nucleotide substitution between population (D_a_)
Comparison at baseline			
Malaria vaccine vs. control group	20.805	0.0172	0.0003
			
Pre- vs. post-immunization			
Control group	20.694	0.0173	0.0004
Malaria vaccine group	20.773	0.0173	-0.0001
			
Comparison after immunization			
Malaria vaccine vs. control group	20.739	0.0172	-0.00002
			
Comparisons with vaccine strains			
At baseline			
Controls vs. 3D7	19.971	0.0193	0.0111
Control group vs. FVO	19.775	0.0173	0.0091
Malaria vaccine group vs. 3D7	21.108	0.0166	0.0078
Malaria vaccine group vs. FVO	21.394	0.0193	0.0104
Post-immunization			
Controls vs. 3D7	20.743	0.0179	0.0111
Control vs. FVO	20.768	0.0193	0.0107
Malaria vaccine group vs. 3D7	20.780	0.0176	0.009
Malaria vaccine group vs. FVO	20.807	0.0192	0.0106

#### Divergence from vaccine strains

*Ama1 *sequences from the vaccine and control groups were compared to the vaccine strains 3D7 and FVO to assess whether infections experienced by individuals receiving the malaria vaccine had sequences that were more divergent from the vaccine strain sequences than those in the control group. All measures of genetic differentiation indicated that immunization with AMA1-C1 had no impact on genetic divergence from the vaccine strains. Both at baseline and following immunization, the vaccine and control group sequences were similarly divergent from FVO and 3D7 based on K, D_xy _and D_a_, suggesting that the vaccine did not increase divergence from the vaccine strains (Table 3).

Additional analyses were performed based only on the polymorphic amino acids in cluster 1 of domain I of AMA1 [10] which includes cluster one loop, to determine whether evidence of selection or reduced diversity would emerge when focusing only on those polymorphisms thought to be most relevant to allele-specific antibody responses [19] and to acquired clinical immunity [16]. Both at baseline and following immunization *ama1 *cluster 1 sequences which includes conserved regions as well as the polymorphic positions ranging from amino acids positions 187 to 230 were similar in the AMA1 vaccine and control groups: At baseline, nucleotide divergence of *ama1 *cluster 1 sequences compared to 3D7 cluster 1 sequence were 0.05374 and 0.0467 for the malaria vaccine and control groups respectively. In infections occurring after immunization, cluster 1 divergence from 3D7 was 0.066 in the AMA1 vaccine group and 0.0530 in control group, respectively with p > 0.1, thus supporting a lack of vaccine selection or allele-specific efficacy. A similar lack of increased divergence from FVO in cluster 1 was observed: nucleotide divergence was 0.0018 in AMA1 vaccine group and 0.0035 in the control group before immunization compared to 0.0013 and 0.0022 in the AMA1 and control group, respectively, following immunization.

The similarity of c1L haplotype distribution in the two treatment arms was further supported when the incidence of infection by haplotype was compared (Table 2). Although there was a lower incidence of clinical malaria caused by 3D7-type AMA1 c1L in the malaria vaccine group (18.6%) compared to the control group (30.8%) following immunization, this reduced frequency did not achieve statistical significance, and there was no suggestion of decreased risk of clinical malaria caused by FVO-type AMA-1 c1L in the malaria vaccine group.

#### Structure analysis

**Figure 3:**
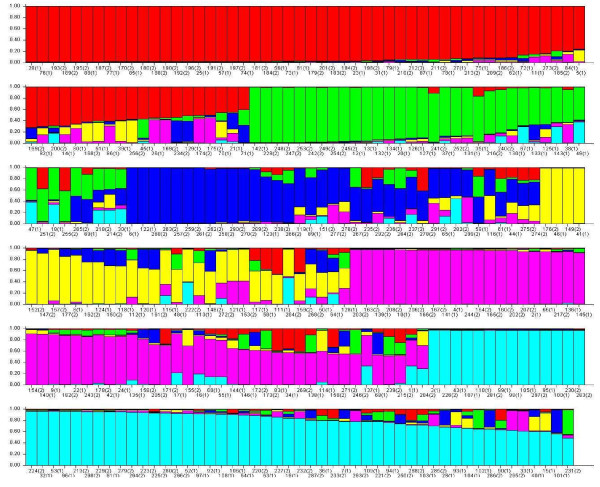
**Population structure of *Plasmodium falciparum *apical membrane antigen-1 (AMA1) sequences using DISTRUCT program **[24]. The population structure of AMA1 was assessed using a Bayesian clustering method implemented in Structure 2.2 with k = 6. Each haplotype is represented by a vertical bar, and each color represents a population. Six populations are shown: Group 1 in red (3D7 group, haplotype 29), group 2 in green (FVO group, haplotype 49), group 3 in dark blue, group 4 in yellow, group 5 in pink and group 6 in light blue.

**Figure 4:**
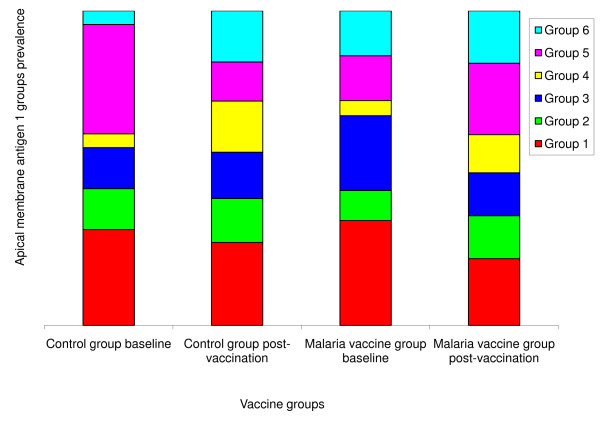
**Distribution of six *Plasmodium falciparum *apical membrane antigen-1 (AMA1) sequence groups before and after vaccination with the malaria vaccine and control vaccine**. Group 1 (red) includes AMA1 sequence corresponding to the 3D7 vaccine strain of *P. falciparum *and Group 2 (green) includes sequence corresponding to the FVO vaccine strain.

Based on results from the incidence study conducted in this site before this vaccine trial [30], we used k = 6 (Figure 3) as the number of groups in the structure analysis. The distribution of sequences in each group is shown in Figure 4 and was used to compute a chi-square test. The test was not significant when the six groups of sequences defined using clustering analysis are compared as follows: malaria vaccine vs. control group sequences at baseline (p = 0.81); control group sequences at baseline vs. after immunization (p = 0.19); malaria vaccine group sequences at baseline vs. after immunization (p = 0.67); and malaria vaccine group sequences after immunization vs. control group sequences after immunization (p = 0.10).

#### Time to first malaria clinical episode with vaccine-type parasites

**Figure 5:**
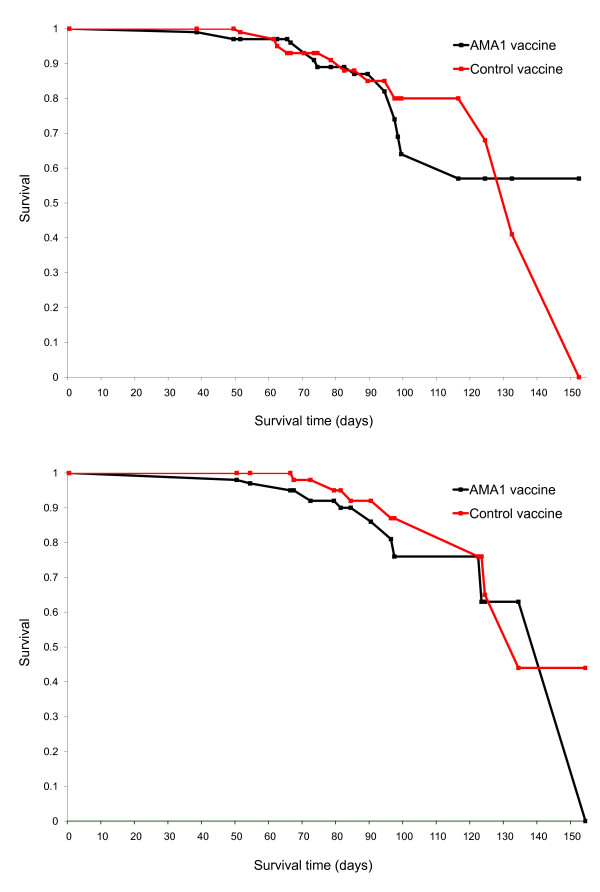
**Kaplan-Meier plot of survival curve without malaria clinical episode with a 3D7 or FVO c1L type allele following vaccination with apical membrane antigen-1 (AMA1) malaria vaccine or a control vaccine**. Top panel, 3D7. Log rank statistic = 0.02, p = 0.88 and Wilcoxon statistic = 0.11 p = 0.73. Bottom panel, FVO. Log rank statistic = 0.35, p = 0.55 and Wilcoxon statistic = 1.70 p = 0.19.

The time to disease with 3D7 and FVO c1L haplotype was also measured. Individuals experienced their first clinical episodes between 6 to 154 days post-immunization (mean 79.2 days). Survival curves (Figure 5A, B) showed no difference between the two groups in time to clinical infection with a 3D7 or FVO c1L haplotype. The occurrence of AMA1 3D7 type clinical episodes in the two treatment arms was identical both at the beginning (log rank test p-value = 0.88) and at the end of the follow up period (Wilcoxon test p-value = 0.73). A similar observation was made when the strain under consideration was FVO (Log rank p-value = 0.56 and Wilcoxon p-value = 0.19).

A Cox proportional hazard regression was performed to model the time to first malaria clinical episode with a c1L haplotype exactly matching the 3D7 or FVO strain as a function of study group. Hazard ratios obtained from the model and vaccine allele-specific efficacy are shown in Table 4. The hazard ratio for clinical illness caused by parasites with 3D7-type AMA1 c1L was 1.06 with a 95% confidence interval (CI) of 0.48-2.32. For FVO the hazard ratio was 1.34 with 95% CI of 0.50-3.62. These results support the conclusion that the AMA1 vaccine and control groups had similar risks of having clinical malaria episodes with parasites similar to the vaccine strains with respect to immunologically important polymorphic amino acid residues.

**Table 4:** Risk of malaria clinical episodes with an apical membrane antigen-1 (AMA1) cluster 1 loop (c1L) haplotype matching vaccine strains

Strain and vaccine group	Hazard Ratio	95% confidence interval	p-value
3D7 strain			
Control vaccine	Reference		
AMA1-C1	1.06	0.48 - 2.32	0.89
FVO strain			
Control vaccine	Reference		
AMA1-C1	1.34	0.50 - 3.62	0.57

## Discussion

The extensive genetic diversity that is maintained in malaria vaccine candidate antigens through balancing selection applied by host immunity may hamper the development of effective malaria vaccines, especially those targeting highly polymorphic blood stage antigens such as AMA1 [35,28]. Molecular epidemiological studies can suggest which antigen variants might best be included in vaccines based on their prevalence in natural populations [16], and molecular epidemiological, population genetics [30] and in vitro invasion inhibition studies [19,36,37] all provide clues about which variants might offer the most cross-protection in multivalent vaccine formulations. However, only field trials of vaccine efficacy against diverse parasites can provide definitive evidence of cross-protection or the lack thereof. Here, results are reported of analyses of allele-specific efficacy of AMA1-C1, a bivalent AMA1 vaccine that was designed to overcome allelic diversity in this extremely polymorphic antigen by including two allelic variants of the target antigen. AMA1-C1 is the first AMA1 vaccine to be evaluated in a field trial measuring efficacy against malaria in a natural setting.

The AMA1 sequences included in AMA1-C1 are derived from the *P. falciparum *strains 3D7 and FVO. These sequences were chosen based on the availability of these two well-characterized culture-adapted strains with divergent sequences, without knowledge of the baseline distribution of the corresponding AMA haplotypes in the natural parasite populations where the vaccine would be tested and eventually deployed. The results of this study show that, based on polymorphisms in the entire AMA1 ectodomain, fewer than 3% of AMA1 sequences examined from samples collected at the vaccine trial site had haplotypes matching 3D7 while none had the FVO haplotypes; very similar results were found at another vaccine testing site in Mali [16]. Thus a possible explanation for the failure of AMA1-C1 to demonstrate protection in a Phase 2 trial in 300 Malian children was that allele-specific immune responses induced by the vaccine, even if highly effective against parasites carrying homologous forms of AMA1 (either with respect to the whole AMA1 ectodomain or some subset of immunologically important epitopes such as c1L on domain I), were not broadly cross-protective enough to result in measurable efficacy against parasitaemia [29]. If an insufficiently broad immune response explained the lack of overall efficacy, allele-specific vaccine-induced immune responses should still have been directed against the fraction of parasites with partial or full homology to 3D7 and FVO AMA1, leading to a reduction in the frequency of these alleles following immunization.

The results of this study provide evidence that insufficient coverage of AMA1 diversity does not explain the lack of vaccine efficacy. Several measures of genetic diversity showed no impact of the vaccine on the diversity of AMA1 alleles in infections experienced by vaccine recipients compared to baseline or to infections in the control group. Moreover, no significant association was seen between vaccination and the risk of malaria clinical episodes caused by parasites with AMA1 similar to the vaccine strain with respect to immunologically dominant regions of the AMA1 protein. There was a non-significant ~1.6-fold lower incidence of clinical malaria caused by parasites with AMA1 c1L haplotypes corresponding to 3D7 following immunization with AMA1-C1 than with a control vaccine (Table 4). However, this hint of possible selection is at odds with the observation of increases in the frequency of 3D7-type c1L in both treatment groups during the post-immunization observation period, and the lack of any suggestion of reduced risk of clinical episodes caused by parasites with FVO-type AMA1 c1L in the vaccine group.

It is more likely that AMA-C1 vaccine failed to protect due to insufficient immunogenicity of the vaccine formulation. For this reason, a new formulation of AMA1-C1 that includes the toll-like receptor agonist CPG 7909 has been developed and tested in Phase 1 trials, which show an approximately 12-14-fold increase in post-immunization antibody levels compared to the formulation without this additional adjuvant in malaria naïve populations, and a 2.5-3-fold increase in antibody levels in malaria exposed adults [38-40]. A monovalent AMA1 vaccine based on the 3D7 strain and formulated with the AS02_A _adjuvant system has also shown strong and sustained antibody responses in Malian adults [41] and children [42]. Results of a Phase 2 safety and efficacy study of this vaccine will be available soon and may allow ascertainment of an allele-specific effect of an AMA1 based malaria vaccine.

Limitations of this study include the possibility that there was insufficient statistical power to detect a subtle degree of allele-specific efficacy. *P. falciparum *infections with AMA1 corresponding to the 3D7 strain with respect to the entire ectodomain represented only 3% of all the haplotypes present at the vaccine trial site at the start of the study. Furthermore, only single or predominant AMA1 sequences could be used to define haplotypes and for population genetic analyses, also contributing to reduced statistical power to detect differences between the malaria vaccine and control groups. To detect a 50% reduction in the frequency of full AMA1 haplotypes by the vaccine, 1,534 unique or predominant AMA1 sequences would have been required to have 80% power with significance at 5%. Statistical power is increased by examining only haplotypes based on the eight polymorphic amino acid positions within the putatively immunologically important region of AMA1, c1L of domain I--to detect a similar effect, 586 unique or predominant c1L haplotypes would be required, demonstrating the challenges of measuring allele-specific efficacy for vaccines based on uncommon antigenic variants. However, the conclusion that the vaccine had no effect on the distribution of AMA1 haplotypes is supported by the near-uniformity of several measures of genetic diversity and divergence from the vaccine strains.

## Conclusion

In this Phase 2 trial of the bivalent blood stage malaria vaccine AMA1-C1 in Malian children, no evidence was found of allele-specific efficacy. AMA1 sequences in infections occurring in the vaccine and control groups were remarkably similar before and after immunization when examined using several complementary statistical and population genetics methods. These results strongly suggest that the AMA1-C1 vaccine failed not due to inability to overcome extensive genetic diversity in AMA1, but for another reason, most likely an insufficiently immunogenic vaccine formulation. Two second generation AMA1 vaccines with more immunogenic adjuvant systems are being evaluated in field trials in Mali. Results of these trials should provide evidence on which to base decisions about whether or not further development of AMA1-based vaccines is warranted, and to guide optimal choices of AMA1 haplotypes to include in a multivalent AMA1 malaria vaccine.

## List of abbreviations

*P. falciparum*: *Plasmodium falciparum*; AMA1: Apical membrane atigen-1; MSP-1: Merozoite surface protein-1; MSP-2: Merozoite surface protein-1; RESA: Ring surface erythrocyte antigen; kDa: kilo Dalton; SPf66, 66 kDa of *P. falciparum *sporozoite protein; ITNs: Insecticide-treated bed nets; RTS,s: Repeat region-T cell epitopes-HbSAg

## Competing interests

The authors declare that they have no competing interests.

## Authors' contributions

**AO **designed the molecular typing study, performed PCR and sequencing analysis, analysed the data, wrote the paper, reviewed the manuscript and approved the final version. **OKD, LHM **and **RDE**, designed the study, coordinated study execution, wrote the report, scientifically reviewed the paper and approved the final draft. **XS **supervised and coordinated the molecular study, reviewed the manuscript and approved the final version. **CVP **supervised and coordinated the molecular study, analysed the data, reviewed and revised the manuscript and approved the final version. **JM **and **ST-H **reviewed the design of the molecular study, analysed the data, reviewed the manuscript and approved the final version. **JD **performed part of the PCR analysis and sequencing, reviewed the manuscript and approved the final version. **RS, AN, AD **and **IS **conducted field study, and approved the final version.
